# Building capacity in horizon scanning, early awareness, and disinvestment: a framework for education and training

**DOI:** 10.1017/S0266462325100354

**Published:** 2025-07-21

**Authors:** Maximilian Otte, Rosmin Esmail, Nora Ibargoyen-Roteta, Iñaki Gutiérrez-Ibarluzea, Hans-Peter Dauben

**Affiliations:** 1 EuroScan International Network e. V, Cologne, Germany; 2 Institut für Gesundheit und Gesellschaft gUG (I2GG), Cologne, Germany; 3 Department of Community Health Sciences, University of Calgary, Calgary, AB, Canada; 4 Acute Care Alberta, Calgary, AB, Canada; 5 O’Brien Institute for Public Health, Calgary, AB, Canada; 6 Basque Office for HTA (Osteba)/Basque Foundation for Health Innovation and Research (BIOEF), Barakaldo, Spain; 7 Health ClusterNET, Amsterdam, Netherlands

**Keywords:** horizon scanning, early awareness, disinvestment, curriculum, education

## Abstract

**Objectives:**

The increasing relevance of horizon scanning (HS), early awareness (EA), and disinvestment (DIS) highlights the need for a structured approach to capacity building. Although these fields are essential for evidence-based policy decisions, a harmonized education and training framework to develop necessary competencies is lacking. This article presents the development of a curriculum designed to address this gap in training.

**Methods:**

A transdisciplinary working group was established, drawing on international stakeholders from academia, the public sector, and industry. Using an iterative consensus-driven approach, the group developed a modular curriculum. The curriculum design incorporated best practices from existing education programs in related fields and emphasized case-based learning strategies to ensure contextual adaptability.

**Results:**

The resulting curriculum covers theoretical foundations, practical applications, and decision-making processes related to HS, EA, and DIS in eight modules. It supports diverse learner needs, including trainees, training institutions, and public and private organizations, and is designed to be flexible, scalable, and applicable across different regional and organizational contexts.

**Conclusions:**

This curriculum initiative represents a major step toward harmonizing capacity building in HS, EA, and DIS. It fosters sustainability, enhances global health system preparedness, and provides a structured educational platform to support the effective integration of emerging health technologies and evidence-based disinvestment strategies.

## Introduction

New and emerging health technologies offer significant opportunities to improve health and well-being (1). They not only have the potential to optimize health outcomes but also to contribute to addressing global challenges as defined in the United Nations Sustainable Development Goals (SDGs) (2). To fully unlock the potential of these technologies, proactive strategies tailored to the specific requirements of different implementation contexts are recommended (3).

A key element of proactive strategies is the early awareness (EA) of emerging technologies and trends (4). EA is linked to processes such as horizon scanning (HS) that provide a structured approach to identify emerging technologies and prioritizes them according to the predetermined decision criteria of different stakeholders and contexts (5). HS describes the “systematic identification of health technologies that are new, emerging, or becoming obsolete and that have the potential to affect health, health services, and/or society” (6).

Disinvestment (DIS) is the “deliberation or systematic reduction of funds for health technologies of questionable or comparatively low value” (7). As health systems integrate new technologies, disinvestment plays a crucial role in ensuring that resources are reallocated effectively, and that outdated or low-value technologies are phased out. This process occurs in tandem with the introduction of new technologies to maintain system efficiency and sustainability (8;9). The redistribution of resources and the modification of established processes require transparent change management to foster acceptance among the actors involved (3;16).

HS, EA, and DIS are closely linked to Health Technology Assessment (HTA) processes. HTA describes “a multidisciplinary process that uses explicit methods to determine the value of a health technology at different points in its lifecycle. The purpose is to inform decision-making to promote an equitable, efficient, and high-quality health system” (11). While HS and EA help to identify promising new technologies early and integrate them into technology development, HTA provides an evidence-based assessment framework to determine their value within a specific setting (e.g., healthcare system). DIS complements this process by systematically reducing, replacing, or eliminating technologies with questionable or low-added value. Together, these approaches form a coherent strategy to optimize resource allocation, technology implementation, and decision making within the health care (10).

Today, EA and HS play a key role in integrating unmet health needs into technology development processes through early dialogue, early advice, and scientific advice (11). This proactive approach can provide invaluable insights for various actors such as health policymakers, regulators, and public and private investors (12). For instance, EA and HS can be instrumental in supporting procurement processes or local production and supply chain strengthening initiatives (13). These initiatives not only consider health-related aspects, but also economic co-benefits, such as regional economic development (14).

Despite the growing importance of HS, EA, and DIS, there is no harmonized platform for education and training that ensures the provision of a structured and synergistic acquisition of the necessary skills in these areas. Initiatives and frameworks such as the EuroScan/i-HTS Toolkit already exist (15–17), however, toolkit’s purpose is to harmonize the HS methodology not to support capacity building (5). There is a lack of overview and consensus on the relevant concepts for education and training in the fields of HS, EA, and DIS.

A harmonized framework that integrates common terminologies and methods could address this gap. The experience of training and e-learning initiatives in the field of HTA – such as HTA 101, the Curriculum Health Technology Assessment, the Ulysses or Validate HTA Project – provides a valuable basis for the development of such training approaches (17–20).

Therefore, two central questions were raised within the HTAi Disinvestment and Early Awareness Interest Group (HTAi DEA IG): (i) How can a basic education and training framework for HS, EA, and DIS be developed? (ii) What required competencies and skills should be integrated into such a framework?

This article addresses these questions and provides a concrete approach establishing an education and training framework for HS, EA, and DIS.

## Methodology

To address the questions posed earlier, two central challenges had to be addressed: (i) the selection of a suitable approach for developing the framework and (ii) the development of a structure for the framework.

### Proposing a curriculum – a suitable approach for framework development

Approaches such as standalone training programs, self-directed learning, or mentorship-based models were initially considered beside curricula. However, these alternatives were found to have significant limitations, including a lack of adaptability to different contexts, inconsistency in knowledge acquisition, reliance on the availability of mentors, and limited scalability for larger groups. Moreover, the absence of a predefined consensus on the essential learning content made these approaches less favorable, as they would have required an established agreement that was not yet in place.

Given these considerations, the decision was made to use a curriculum as the primary instrument for structuring learning. A curriculum offers a coherent, step-by-step learning experience, facilitates consensus among stakeholders on essential content and quality standards, and allows for iterative development to ensure ongoing relevance and acceptance. Furthermore, a modular curriculum provides flexibility and adaptability, enabling the selective tailoring of content to different contexts, learning levels, and prior knowledge (21).

### Target audiences of the curriculum

The different application environments of HS, EA, and DIS described earlier result in three target audiences for a curriculum:

1. Trainees: Individuals seeking to acquire, update, or certify knowledge and skills in HS, EA, and DIS.
2. Training institutions: Organizations aiming to integrate the curriculum into their programs or develop targeted courses based on its content.
3. Public and private organizations: Entities using the curriculum for internal capacity building, decision making, and policy development in health technologies and resource management.

**Figure 1.**
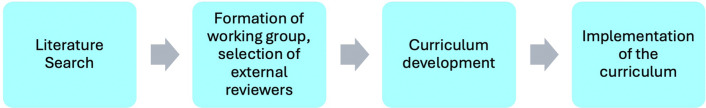
Step-by-step process for developing and implementing the curriculum.

Figure 1 describes the process in the development of the framework.

### Methods to develop the framework

#### Step 1: literature search

To recruit experts for a working group to develop a curriculum, a preliminary literature search was carried out. For this purpose, a narrative literature search was performed between June and July 2021 in Google Scholar, PubMed, Scopus, Web of Science, and Embase to gain an initial overview of the following topics:

- Concepts and methods related to HS, EA, and DIS.
- Curriculum development approaches from the field of life sciences, especially in the context of health research and technologies.

Relevant search terms were “horizon scanning,” “early awareness,” “disinvestment,” “health technology assessment,” “curriculum development,” “education,” “training,” and “health technology decision making.” From these terms, a snowball search was conducted.

The results of the search were summarized and made available to the members of the DEA IG for review. The search provided the evidence for the next steps.

#### Step 2: formation of the working group and selection of external reviewers

A transdisciplinary working group was set up in August 2021. The primary objective of the working group was to establish a consensus on content across organizational boundaries. EuroScan/i-HTS and HTAi involve stakeholders from academia, the public sector, and – in the case of HTAi – industry. To ensure a broad, global perspective, the following selection criteria were considered:

- Expertise in HS and related fields such as HTA, health economics, or health technology management.
- Practical experience in the application of HS or academic training in relevant fields.
- Geographic representation from different regions of the world to include health policy, societal, cultural, and economic contexts.

External reviewers, independent of the working group, contributed to the iterative refinement of the curriculum to enhance its quality and ensure its relevance. The reviewers were selected using the same criteria as the working group to ensure methodological rigor.

#### Step 3: curriculum development

The curriculum development process was based on previous approaches used in curriculum development from the life sciences. Several approaches were considered (20–24). The approach by Schneiderhan et al. was identified as suitable and served as the basis for the further development process (24). This approach was considered best suited to achieve cross-organizational consensus and harmonization between disciplines. The structured, step-by-step methodology was developed specifically for medical and health-related education. The approach includes a needs assessment phase in which input is gathered from a range of stakeholders and requirements are defined for different user groups to ensure that the curriculum meets the requirements of the target groups. Schneiderhan et al. also highlights good practice in formulating learning objectives to ensure high-quality educational outcomes. This enabled the transdisciplinary working group to quickly develop a common methodological basis and understanding to work together effectively.

### Requirements analysis and definition of training objectives

An initial workshop of the working group was held to analyze the requirements of the target groups described. Based on the requirements analysis, specific learning objectives and competence fields were developed. An overarching learning objective was formulated for each identified area of expertise. In addition, the expectations of the learners were specified by describing exactly which skills and knowledge they should have acquired after completing the modules.

The decision to conduct a requirements analysis instead of a needs analysis was based on the pre-existing evidence and expert consensus on the necessity of structured training (25–32). Previous studies, policy reports, and international initiatives – such as the Toolkit of EuroScan/i-HTS – had already highlighted the lack of a standardized educational framework in these areas (5). Consequently, the focus was placed on defining the precise competencies and learning objectives required to address the identified gaps.

### Content development and prioritization

The content was developed and prioritized in an iterative process. Two experts from the working group initially drew up an initial overview of areas of expertise based on the formulated requirements and training objectives, which included both theoretical knowledge (e.g., about the life cycle of health technologies (33) and practical knowledge (e.g., project and knowledge management tools). This overview was discussed in a second workshop within the working group. The refined and agreed upon overview was then externally reviewed by five representatives from Malaysia, Switzerland, Norway, and Australia. These reviewers, who were not part of the Working Group, represented various members of EuroScan/i-HTS and several HTAi Interest Groups and were affiliated with organizations identified as target entities for the curriculum. Following the review, the overview was expanded to include detailed descriptions of each competency area, with each area presented as a module featuring a title, educational goal, and content. This expanded version was shared with the external reviewers, and in a final workshop, their suggestions were addressed, leading to the approval of the final curriculum by the working group.

In the content development process for each module, the content was proposed, discussed, and where possible, agreed by consensus. In the event of a lack of consensus, provisional content was included in the curriculum. The final decision was then made after the external review.

### Selection of training strategies

As part of the creation process, various learning strategies were considered according to Schneiderhan et al., including lecture-based information delivery, hands-on skill delivery, the flipped classroom approach, and case-based lectures (24). Ultimately, the working group identified the case-based learning approach as the most suitable, as it promotes practical learning environments and enables contextualization to regional or institutional requirements. Such case studies can be derived from real application environments and can be integrated into training units, thus ensuring direct relevance for the learners.

#### Step 4: implementation of the curriculum

To promote the acceptance and relevance of the curriculum as part of the implementation process, official endorsement was sought and granted by EuroScan/i-HTS and HTAi. This endorsement serves to strengthen the credibility of the curriculum and underline its value. In parallel, the resources required to implement the curriculum were identified. Key resources included qualified individuals to act as lecturers and facilitators of the content, and institutions willing to use the content of the curriculum in their specific contexts. Additionally, funding was considered necessary for the development of interorganizational e-learning offerings to accelerate the practical application of the curriculum content in diverse real-world educational and professional settings.

Based on this requirement analysis, the next step was to specifically request external support. To initiate the implementation process, a joint introductory webinar by EuroScan/i-HTS and HTAi was identified as an appropriate format to introduce the general approach and curriculum content to a wider audience. The goal was to identify potential lecturers, experts from EuroScan/i-HTS, HTAi, and related professional societies involved across the lifecycle of health technologies (33).

These individuals, recognized for their expertise in relevant fields, would be recruited to lead a series of topic-specific webinars jointly hosted by EuroScan/i-HTS and HTAi through the DEA IG. The webinar series would not only serve as the foundation for the development of e-training courses, but also raise broader awareness of the new curriculum among target audiences.

To offer e-learning courses, an electronic learning management system was investigated. Such a system would enable flexible learning opportunities and provide a sustainable platform for the continuation and scaling of curriculum content based on common standards such as SCORM (Sharable Content Object Reference Model). SCORM enables the creation, management, and exchange of electronic learning content and courses in learning management systems, facilitating the reusability of e-learning content across different platforms (34;35).

In addition, the curriculum was to be presented at international conferences (e.g., HTAi Annual Meeting, HTAsiaLink Annual Conference) as part of workshops designed to conduct beta testing of the curriculum content with different stakeholders in various contexts. This was done to identify potential barriers to implementation.

## Results

### The curriculum

The working group finalized the curriculum structure by clustering and organizing the content developed during the workshops into eight modules. Each module includes clearly defined educational goals and specifies the skills learners are expected to acquire upon completion. Table 1 provides an overview of each module. The full curriculum can be downloaded from Supplementary File 1.

**Table 1.** Extract from the curriculum: areas of expertise

Area of expertise	Subordinate educational goal	Skills to be acquired
1. Basic principles – life cycle of a technology	Understanding the integrative approaches of the different tools and methods as well as scientific concepts within the framework of life sciences, technology life cycle, and health	Background to development of the life cycle concept, knowledge about common terminology
2. The health technology development framework – from needs to technologies in action	Supporting the developers on their way to achieving a technology for usage within the area of health Understanding of the life-cycle concept of technologies and related activities	Knowledge about different health needs and their metamorphosis into addressing technologies, influencing factors, and stakeholders involved
3. Health determinants and user groups within the different steps of horizon scanning	Understanding of importance of multidimensionality and multidisciplinary in value determination of health technologies per se and in relation to different user groups as well as to understand the domain concept in relation to a structured comparison of technologies	Knowledge about how to align horizon scanning activities with addressed health determinants and user groups
4. Modelling and the estimation of the future activities	Understanding that methods must be constantly modified to meet the requirements of the field of work. Other research disciplines can also provide methods that may be used	Knowledge about decision-analytical modeling, its areas of application, and critical appraisal
5. Knowledge and project management competences	Understanding the importance and advantages of systematic approaches and the development of strategies in the project preparation phase and beyond	Knowledge about best practices in systematic and reliable approaches, building trust in results
6. Quality assurance	Understanding the role and importance of different quality assurance processes in preparing high-quality evidence in various settings	Knowledge about ensuring and evaluating quality of evidence and the technology
7. Communication and dissemination	Developing an understanding that the way information should be disseminated depends on the target group, its background, and information needs	Knowledge about the involvement of target groups, dealing with different knowledge backgrounds
8. Scientific tools and methods	Learning about specific methods and instruments of the subject area	Knowledge about quantitative and qualitative approaches and methods to describe technologies and their implications

### Initiating the implementation process

Following the official endorsement of the curriculum by EuroScan/i-HTS and HTAi in January 2022, the structured implementation process began. A key milestone was the joint EuroScan/i-HTS and HTAi webinar on 16 March 2022, in which the background, content, and objectives of the curriculum were presented. Representatives from EuroScan/i-HTS, HTAi, and members of the DEA IG presented the key elements and encouraged active participation. This webinar was recorded and made publicly available via the EuroScan/i-HTS website (provide link) (36).

The introductory webinar was followed by a series of topic-specific webinars led by experts in the respective fields. These webinars covered a broad spectrum, ranging from an introduction to HTA and hospital-based HTA to specific topics such as bioethics, advanced therapy medicinal products, and sustainability in scientific evaluation. Between April 2022 and February 2023, a total of 10 joint webinars were held by EuroScan/i-HTS and HTAi. These events were also recorded and made available online to provide retrospective accessibility (https://ihts.org/webinars/).

In parallel, the content of the curriculum was presented at international conferences and in workshops. These included workshops at the 2022 HTAsiaLink conference in Pattaya (Thailand), the 2023 HTAi annual conference in Adelaide, and the 2023 HTAsiaLink conference in Putrajaya (Malaysia) (37–39). Further training activities took place at the RedETSA conference in November 2024 and the SCAN 2023 initiative in Hong Kong in December 2024 (40;41). These events not only offered the opportunity to present the curriculum to an international audience, but also served to gather feedback from stakeholders and continuously evaluate the content.

In addition, the evidence.academy was established in 2023 as an e-learning platform for training courses on HS, EA, and DIS. To maintain it as a sustainable solution for a broad community, it was launched using a Moodle-based approach (www.evidence.academy). Moodle offers a cost-effective and customizable e-learning platform with a high degree of flexibility, integration options, and support from an active open-source community (42).

## Discussion

This article describes the successful development of a modular curriculum for HS, EA, and DIS. It provides a structured basis for teaching the skills required to apply these fields in different contexts.

The curriculum comprises eight modules covering both theoretical knowledge and practical skills. These include basic concepts related to the life cycle of health technologies (33), as well as the modeling of potential effects of emerging health technologies on health, health systems, and society (5). The curriculum also covers areas of expertise in knowledge, project, and quality management, target group-oriented communication, and the application of specific techniques and methods for evidence-based decision support.

The curriculum approach uses elements of existing approaches from Perleth et al. in the field of HTA. This includes, the modular structure of learning content, the alignment of learning content within the modules to overarching learning objectives, and the inclusion of case-based learning for the integration of practice-relevant problems into the training, also in connection with e-learning programs. Furthermore, the development and maintenance of the curriculum is also seen as an interorganizational task to ensure relevance and acceptance. The curriculum is novel as it describes a global perspective and can be applied in all jurisdictions rather than the requirements in a specific language or context (20). It is, thus, flexible and generic.

The curriculum addresses a critical gap in training, education, and capacity building. Furthermore, it offers perspectives that extend beyond the mere teaching of specialized skills or the training of specialists. By fostering a basic understanding of HS, EA, and DIS, it cultivates a broader societal perspective on the potential implications of emerging technologies which not only can drive improvements in health outcomes but also can tackle key societal challenges such as wealth distribution and sustainable development (2).

Furthermore, the curriculum integrates discussions on DIS, encouraging a dialog on the necessity of phasing out outdated practices and low value care for innovative, evidence-based approaches (36).

The main limitation is the lack of practical application of the curriculum in different contexts, user groups, and organizations. There have been initial evaluations of the curriculum in webinars and at conference, but a systematic evaluation with specific user groups and organizations is still pending. This is particularly important with regard to acceptance and applicability in different regional and cultural contexts (3) and will be considered in the next phase of its implementation.

## Conclusion

The curriculum marks a significant advancement in addressing educational gaps in HS, EA, and DIS. By drawing upon proven methodologies and aligning with global health priorities, it provides a structured, flexible, and context-sensitive framework for building capacities. The collaborative approach used in its development supports its relevance and adaptability, paving the way for adoption and spread. With ongoing refinement and implementation, the curriculum holds the potential to transform capacity-building efforts in these critical areas, ultimately contributing to better health outcomes and sustainable health systems.

## Supporting information

Otte et al. supplementary material Otte et al. supplementary material

## Supplementary material

The supplementary material for this article can be found at http://doi.org/10.1017/S0266462325100354.
