# Supplementary material for: Building capacity in horizon scanning, early awareness, and disinvestment: a framework for education and training
Source: Int J Technol Assess Health Care. 2025 Jul 21;41(1):e51. doi: 10.1017/S0266462325100354 (PMC12322852; doi:10.1017/S0266462325100354)
Supplement: Otte et al. supplementary material [file S0266462325100354sup001.pdf]

Curriculum on Horizon Scanning

Early Awareness (EA) & Disinvestment (DIS)

The scientific approach from the identification, filtration and prioritisation of new and emerging technologies to the Identification and assessment of technologies of no or low-added value in Health Service Areas

Background

Early awareness and Disinvestment of health technologies are increasingly gaining interest globally with rapid evolvement in innovation and technology development. Within different networks related to technology assessment such as international HealthTechScan (i-HTS) and Health Technology Assessment international (HTAi) common methods and techniques are being established and more are under development. Nevertheless, there is currently no framework for structured education and training in this area, and this is increasing the misunderstanding of the actual tools and concepts. i-HTS and HTAi have collaborated to develop and maintain a shared curriculum on Horizon Scanning. Horizon Scanning includes Early Awareness as well as disinvestment activities. The purpose of this curriculum is to teach these concepts and certify organizations using this curriculum to ensure they are following the same standards. Both organisations will be providing the training within the scientific and academic platforms on Early Awareness and Disinvestment in health care services.

Goals for target audiences:

Trainees	Training organisations	Industry and Public institutions
• To be able to provide certification on HS and Disinvestment<br>• To have learnt about EA and Disinvestment<br>• To update knowledge and skills	• University / training organizations to integrate curriculum in their education<br>• Training institutions offering specific courses on EA and Disinvestment as a full time or part time training course	• Support capacity building in EA and Disinvestment<br>• Supporting decision makers in health care, life-science and technology development areas by training in specific skills in relation to health care technologies<br>• Supporting staff training in EA and Disinvestment

Due to these varied target audiences, the curriculum will be an overall document describing the content framework. In addition, the different target audiences will require more specific skills and training specific for certification and overall mutual recognition of training.

The curriculum will include different scenarios describing the content and the referred skill requirements of the trainees, the trainers, and the trainings institutions.

The framework of training

Area of expertise	Subordinate educational goal	Skills to be acquired
1. Basic principles - life cycle of a technology	Understanding the integrative approaches of the different tools and methods as well as scientific concepts within the framework of life sciences, technology life cycle and health	Background to development of the life cycle concept, Knowledge about common terminology
2. The health technology development framework – From needs to technologies in action	Supporting the developers on their way to achieving a technology for usage within the area of health. Understanding of the life-cycle concept of technologies and related activities	Knowledge about different health needs and their metamorphosis into addressing technologies, influencing factors and stakeholders involved
3. Health Determinants and user groups within the different steps of Horizon Scanning	Understanding of importance of multidimensionality and multidisciplinary in value determination of health technologies per se and in relation to different user groups as well as to understand the domain concept in relation to a structured comparison of technologies	Knowledge about how to align Horizon Scanning activities with addressed health determinants and user groups
4. Modelling and the estimation of the future activities	Understanding that methods must be constantly modified to meet the requirements of the field of work. Other research disciplines can also provide methods that may be used	Knowledge about decision-analytical modelling, its areas of application and critical appraisal
5. Knowledge and project management competences	Understanding the importance and advantages of systematic approaches and the development of strategies in the project preparation phase and beyond	Knowledge about best practices in systematic and reliable approaches, building trust in results
6. Quality assurance	Understanding of importance of different quality assurance procedures in preparing high quality evidence in various settings	Knowledge about ensuring and evaluating quality of evidence and the technology
7. Communication and dissemination	Developing an understanding that the way information should be disseminated depends on the target group, its background and information needs	Knowledge about the involvement of target groups, dealing with different knowledge backgrounds
8. Scientific tools and methods	Learning about specific methods and instruments of the subject area	Knowledge about quantitative and qualitative approaches and methods to describe technologies and their implications

Areas of competences in detail

1. Basic principles - life cycle of a technology

Superordinate education goal:
Understanding important concepts, tools, and methods as well as integrative approaches related to health, horizon scanning und health technologies.

Content:
- The concepts of health, diseases, and health determinants
- The concept of evidence-based medicine
- The life-cycle concept of technologies
- Different scientific approaches for decision support during the life cycle of a technology:
  Health-in-all-policies (HIAP),
  Health Needs Assessment (HNA),
  Early Awareness including Scientific Support (EA),
  Health Impact Assessment (HIA),
  Health Technology Assessment (HTA) and Appraisal,
  Health Technology Management (HTM) and
  Disinvestment and obsolete technologies (DIS)

2. The health technology development framework – From needs to technologies in action

Superordinate education goal:
Supporting the developers on their way to achieving a technology for usage within the area of health. Understanding of the life-cycle concept of technologies and related activities

Content:
- Health needs and prioritization
- Technology development and scientific research
- Early awareness, Early advice, Early dialogue
- Investment in development
- Health impact: modelling the impact in different domains
- Disinvestment and rational handling of obsolete technologies in consideration of internal barriers that hamper exclusion of low-value technologies

Additional concepts in **relationship to Horizon Scanning:**
- Sustainability and Innovation as concepts within the scientific assessment
- Comparing technologies and integration in regulatory and reimbursement
- Guidelines and training, appropriate use
- Implementation and management of technologies

3. Health Determinants and user groups within the different steps of Horizon Scanning

Superordinate education goal:
Understanding the importance of multidimensionality and multidisciplinary approaches in value determination of health technologies per se and in relation to different user groups, as well as to understand the domain concept in relation of a structured comparison of technologies.

Content:
- Health aspects and disease impact

- Safety (patient, users, and staff)
- Ethics and cultural aspects
- Social aspects and impact on the social environment
- Legal aspects
- Economic and Health Economic aspects
- Environmental aspects (production, usage/logistics, waste)

4. Methods of modelling and the estimation of the future/what the future looks like

Superordinate education goal:
Understanding different methods that can be used as well as necessity of needed modifications of methods to meet requirements of field of work. Other research disciplines can also provide methods that may be used.

Content:
- Modelling of data
- Combining qualitative and quantitative research
- Different approaches in acquiring scientific data
- Validating scientific data in relation to decision questions
- Usage of data, digital biomarkers and artificial intelligence within scientific analysis
- Real-World Data and Real-World Evidence

5. Methods of knowledge and project management

Superordinate education goal:
Understanding the importance and advantages of team and systematic approaches and the development of strategies in project preparation phase and beyond.

Content:
- Classification and terminologies
- Team approaches in transdisciplinary groups
- Project management
- Workflow and processes
- Knowledge management,
  Knowledge transfer and
  Knowledge translation

6. Methods of quality assurance

Superordinate education goal:
Understanding of importance of different quality assurance procedures in preparing high quality evidence in various settings.

Content:
- internal quality assurance procedures
- Legal requirements of an appraisal and external quality assurance procedures
- Process management and standard operating procedures (ISO)
- Data handling and data quality (ISO)
- Sustainability (ISO)

7. Methods of communication and dissemination
Superordinate education goal:
Developing and understanding that the way information should be disseminated depends on the target group, its background and information needs/purpose.

Content:
- Localizing evidence and needs with specific user groups/stakeholders
- Identifying and including different user groups
- Communication between different scientific disciplines
- Change management
- Risk and uncertainty communication

8. Tools and methods in science

Superordinate education goal:
Learning about specific methods and instruments of the subject area.

Content:
- Filtration and prioritization
- Critical appraisal tools
- Workflow management
- Ontologies and databases
- EA tools
- DIS tools

References

Haas, Sabine; Busch, Martin; Kerschbaum Johann; Türscherl, Elisabeth; Weigl, Marion (2012): Health in All Policies am Beispiel der die Kinder- und Jugendgesundheit (Wissen 9), hg. v. GÖG/FGÖ. Gesundheit Österreich / Geschäftsbereich Fonds Gesundes Österreich. Wien

Matthias Perleth, Kurt Bestehorn, Reinhard Busse, Ansgar Gerhardus, Dagmar Lühmann, Victor Paul Meyer, Uwe Siebert, Curriculum Health Technology Assessement, 2005, Verein zur Förderung der Technologiebewertung im Gesundheitswesen (HTA) e. V., Fachbereich HTA im Deutschen Netzwerk Evidenzbasierte Medizin

Gutiérrez-Ibarluzea I, Chiumente M and Dauben H-P (2017) The Life Cycle of Health Technologies. Challenges and Ways Forward. Front. Pharmacol. 8:14. doi: 10.3389/fphar.2017.00014

EuroScan international network e. V. (2018) Toolkit for identification, prioritisation, filtration and assessment of new and emerging health technologies

Perleth M, Schnell-Inderst P, Rüther A, Raatz H, Kolominsky-Rabas P, Panteli D, Siebert U, Berndt N, Wahlster P. Das Curriculum Health Technology Assessment (HTA), Version 2.0. GMS Health Technol Assess. 2017;13:Doc03. DOI: 10.3205/hta000129, URN: urn:nbn:de:0183-hta000129

Hofmann, B. (2021). Internal barriers to efficiency: Why disinvestments are so difficult. Identifying and addressing internal barriers to disinvestment of health technologies. Health Economics, Policy and Law, 16(4), 473-488. doi:10.1017/S1744133121000037

List of authors and contributors

**Maximilian Otte**<br>**Hans-Peter Dauben**<br>**Izzuna Mudla Ghazzali**<br>**Christoph Künzli**<br>**Inaki Guiterrez Ibarluzea**	**International HealthTechScan (i-HTS)**
**Rosmin Esmail,**<br>**Anna Lien Espeland**<br>**Janet Wale**<br>**Bjørn Morten Hofmann**	**HealthTechnologyAssessment international (HTAi)**

In addition, the networks had invited their members for comments. We thank EuroScan int.net / i-HTS members and the HTAi IG DEA for their contributions.

Remarks

All technical terms that were used in this curriculum are described in two HTA related glossaries available on http://htaglossary.net/Homepage and https://glossary.ihts.org.

Document history:

Version 0.4	first version for endorsement
Version 1.0	first public version
Version 1.0.1	Minor corrections, contributors and design
